# Improvement in diagnostic quality of structural and angiographic MRI of the brain using motion correction with interleaved, volumetric navigators

**DOI:** 10.1371/journal.pone.0217145

**Published:** 2019-05-17

**Authors:** Mads Andersen, Isabella M. Björkman-Burtscher, Anouk Marsman, Esben Thade Petersen, Vincent Oltman Boer

**Affiliations:** 1 Danish Research Centre for Magnetic Resonance, Centre for Functional and Diagnostic Imaging and Research, Copenhagen University Hospital Hvidovre, Hvidovre, Denmark; 2 Philips Healthcare, Copenhagen, Denmark; 3 Department of Radiology, Clinical Sciences Lund, Lund University, Lund, Sweden; 4 Department of Medical Imaging and Physiology, Skåne University Hospital, Lund, Sweden; 5 Lund University Bioimaging Centre (LBIC), Lund University, Lund, Sweden; 6 Centre for Magnetic Resonance, Department of Health Technology, Technical University of Denmark, Kgs. Lyngby, Denmark; Brigham and Women’s Faulkner Hospital, UNITED STATES

## Abstract

**Introduction:**

Subject movements lead to severe artifacts in magnetic resonance (MR) brain imaging. In this study we evaluate the diagnostic image quality in T1-weighted, T2-weighted, and time-of-flight angiographic MR sequences when using a flexible, navigator-based prospective motion correction system (iMOCO).

**Methods:**

Five healthy volunteers were scanned during different movement scenarios with and without (+/-) iMOCO activated. An experienced neuroradiologist graded images for image quality criteria (grey-white-matter discrimination, basal ganglia, and small structure and vessel delineation), and general image quality on a four-grade scale.

**Results:**

In scans with deliberate motion, there was a significant improvement in the image quality with iMOCO compared to the scans without iMOCO in both general image impression (T1 p<0.01, T2 p<0.01, TOF p = 0.03) and in anatomical grading (T1 p<0.01, T2 p<0.01, TOF p = 0.01). Subjective image quality was considered non-diagnostic in 91% of the scans with motion -iMOCO, but only in 4% of the scans with motion +iMOCO. iMOCO performed best in the T1-weighted sequence and least well in the angiography sequence. iMOCO was not shown to have any negative effect on diagnostic image quality, as no significant difference in diagnostic quality was seen between scans -iMOCO and +iMOCO with no deliberate movement.

**Conclusion:**

The evaluation showed that iMOCO enables substantial improvements in image quality in scans affected by subject movement, recovering important diagnostic information in an otherwise unusable scan.

## Introduction

Subject movement is a major problem in magnetic resonance (MR) brain imaging, as the patient is often required to lie still over the several minutes that are required for scanning[1]. Movement may occur in all patient groups, but it happens particularly often in children and the elderly, in patients who are anxious or have pain, and in patients with neurodegenerative diseases [2,3]. Motion leads to reduced diagnostic quality and the need for re-scanning, with prolonged examination times or rescheduling of patients in clinical practice. In research studies, motion reduces the quality and consistency of data. In a retrospective study of one week of clinical MR examinations at their institution, Andre et al. [1] found that 15.4% of the head, brain, and neck examinations had motion artefacts that led to marginal or non-diagnostic quality.

In contrast to other body parts, the head is well approximated by a rigid body. As long as the movement of the head can be accurately quantified in some way, so-called prospective motion correction can be performed by updating the angulations and translations of the imaging volume during scanning to follow the position of the head [4]. The required real-time information on the head position can be obtained in several ways, for example using cameras [5]or magnetic field sensors [6,7], or with purely MR-based navigators where low-resolution MR images or strategic k-space trajectories are acquired in the dead time of the target imaging sequence. The MR-based navigators are especially appealing in the clinical setting, as they have shown high accuracy, but they do not require additional hardware or patient preparation for rigid fixation of markers or sensors on the patient.

Several navigator schemes have been proposed[8–13], and the image-based navigators in particular, such as the low-resolution 3D echo-planar imaging (EPI) volume navigator “vNAV”[14], have proven to be very powerful [15–18]. In this study, we used a similar, flexible framework for prospective motion correction with volumetric navigators (dubbed iMOCO). While in previous conference contributions iMOCO has also been demonstrated in spectroscopy and arterial spin labeling sequences [19,20], in the present study we more rigorously evaluated its use in a T1-weighted, a T2-weighted, and an angiography sequence. For the T1- and T2-weighted sequences, a vNAV-like navigator was used whereas a fat-selective, segmented navigator was used for the angiography sequence. Experiments with different motion scenarios were performed in healthy volunteers, and an experienced neuroradiologist evaluated the diagnostic image quality.

## Materials and methods

### Motion correction framework and reacquisition

Motion correction was performed in all sequences by using a recently developed interleaved scanning architecture [21] which allows instantaneous switching between two (or more) scans at any time point during the execution of the scan. The navigator and target sequence were defined as two subsequent sequences that could be modified in the user interface and interleaved at e.g. every repetition time (TR), slice or volume. During scanning, the latest acquired navigator volume was registered to the first in the series, and translation and rotation parameters of both the navigator and target sequence were correspondingly updated in real time.

As the motion updating is performed with gaps in the order of seconds, the k-space segments acquired in-between motion updates are likely to be corrupted by motion. Reacquisition of a k-space segment in the target sequence was therefore performed when the amount of motion detected exceeded a specified threshold. Reacquisition causes an increase in scan time, but we observed additional improvements in image quality by using reacquisition. For thresholding, a motion score parameter was adapted from Tisdall et al. [14], which calculates the largest displacement on the surface of a sphere with a radius of 64 mm. A threshold of 1 mm of equivalent motion was used for reacquisition in all experiments.

### Integration into target sequences

#### T1-weighted imaging

**Fig 1:**
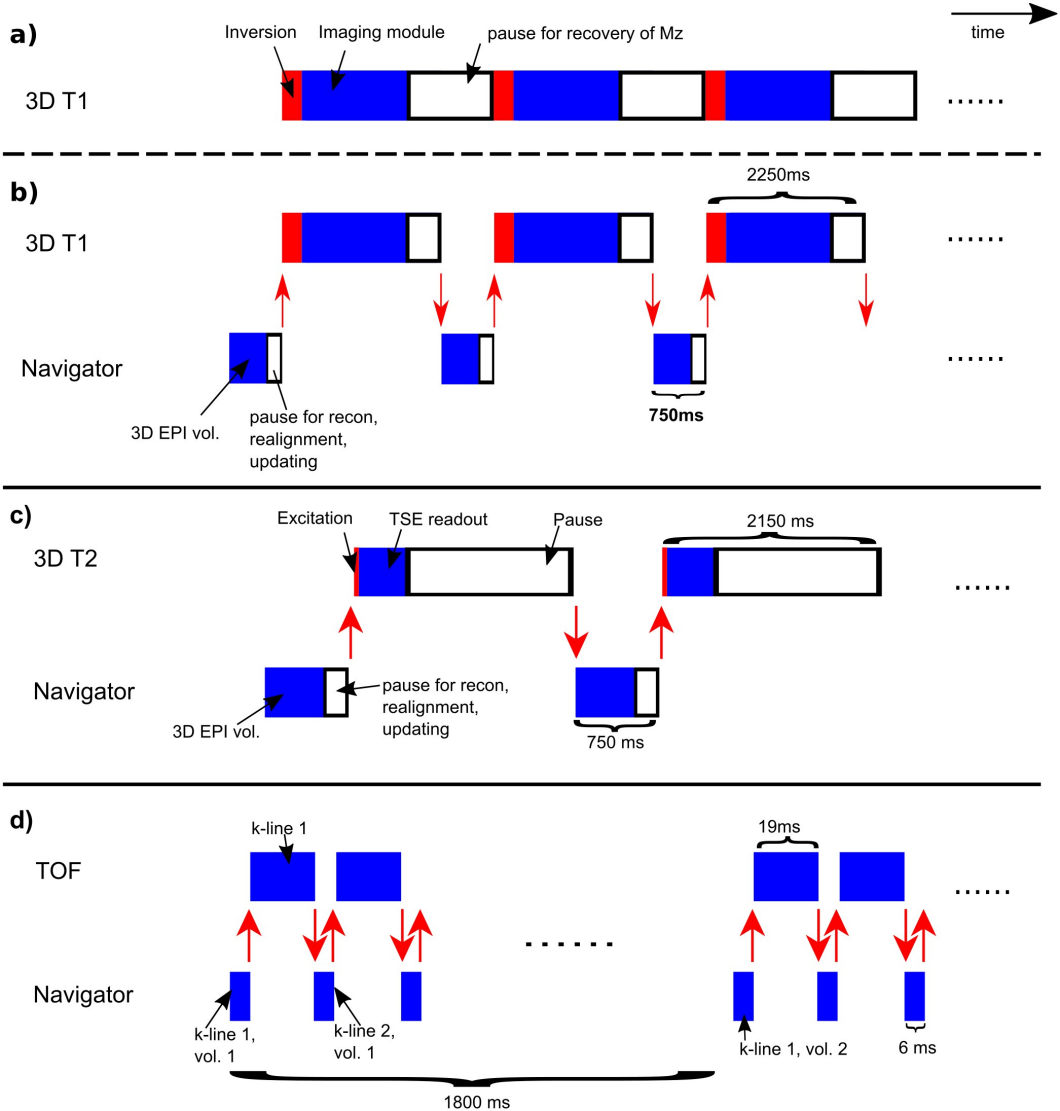
Overview of the switching between host sequences and navigators with associated timings. **a**. The 3D T1-weighted sequence without an interleaved navigator. **b**‒**d**. Sequence layouts after incorporation of navigators. In all cases, the host sequence and navigator were defined as two different sequences in the standard user interface. The red arrows represent the switch between sequences.

As navigator, a 3D EPI sequence was used. Resolution = 7 × 7 × 8 mm^3^, tip angle = 2°, similar to the vNAV [14]. The navigator was interleaved in a T1-weighted 3D MPRAGE of isotropic resolution of 0.85 mm and scan duration of 6 min (Fig 1a and 1b). In the T1-weighted sequence, the relaxation delay before every inversion pulse was shortened by the volume repetition time of the navigator. The switch between scans was performed after the relaxation delay in the T1-weighted sequence and after every volume repetition of the navigator, such that one motion update was available before every inversion pulse in the T1-weighted sequence.

#### T2-weighted imaging

The same navigator as used for the T1-weighted sequence was interleaved with a 3D T2-weighted turbo spin-echo (TSE) sequence with isotropic resolution of 0.85 mm and scan duration of 8 min (Fig 1c). Here, the navigator was placed in the long relaxation delay after each TSE readout, in order to have a motion update just before every excitation.

#### Time-of-flight (TOF) angiography

The TOF sequence was set up as a 3D gradient echo sequence with resolution = 0.25 × 0.49 × 1 mm^3^, scan duration = 3 min, TR = 25 ms and tip angle = 20°. As the steady-state tissue signal in a TOF sequence is highly suppressed; neither much tissue signal nor dead time is available to perform a navigator sequence. A lipid-selective fat-navigator [13] was therefore implemented as a 3D gradient echo with parallel imaging acceleration SENSE factors of 4 × 2, tip angle = 2°, resolution = 7 × 7 × 8 mm^3^. One TR of the segmented navigator was interleaved with one TR of the TOF sequence, providing a full navigator volume every 1.8 s (Fig 1d). As a pause for reconstruction and realignment of the navigators every 1.8 second would disturb the steady state of the TOF, no such pause was inserted, and the geometry updating was instead performed after one more full navigator volume was acquired, so with a delay of 1.8 s. In case the reacquisition threshold was exceeded, the whole 3.6 s segment was reacquired from starting the navigator acquisition to the point of motion updating.

### Experiments

All experiments were performed with a 3T MR scanner (Achieva; Philips Healthcare, Best, the Netherlands) and a 32-channel receive head coil. The study was approved by the Ethics Committee of the Capital Region of Denmark and performed according to the directives of the Declaration of Helsinki (amendment of Fortaleza, 2013). All participants provided written informed consent prior to the examination.

The prospective correction of the three sequences was tested in eight healthy volunteers (four woman and four men, aged 26‒55) by acquisitions with and without deliberate head movement, where the prospective motion correction of the target sequence was turned once on (+iMOCO) and once off (-iMOCO), resulting in four scans per sequence. Due to scan time restrictions, the scans without motion with correction were only collected in 5 out of 8 subjects. To reproduce motion across the scans with and without correction, the subjects were asked to move on command at 5‒8 fixed time points during the scans depending on sequence type and duration. Four of the subjects were asked to perform “stepwise” motion where a new position was held after each movement. Two subjects were asked to perform “jerks”, where the head was rapidly moved to a new position and then immediately back, and the last two subjects were asked to perform “slow jerk motion” where the head was moved slowly and then back to its initial position. Both nodding movements (head rotation around the left-right axis) and shaking movements (head rotation around the superior-inferior axis) were performed in all scans with deliberate motion. The subjects were carefully instructed how to perform these motion patterns. They were kept blind regarding in which scans iMOCO was activated or not activated, and the order was varied across subjects and sequence types.

### Evaluation of image quality and statistical analysis

Diagnostic image quality was evaluated by a senior neuroradiologist with more than 20 years of MR experience. Scoring was randomized, and the reader was blind regarding subjects, deliberate movement, the type of motion, and whether iMOCO was used. An existing 4-grade scale was used for image assessment (Table 1) that is comprised of poor image quality, fair image quality, good image quality and excellent image quality. Note that scores of excellent image quality are giving to a scan that is too good for it’s purpose, and is used in sequence optimization to identify scans that can be reduced in scan time (MR) or dose (CT). This rating system was used to grade 11 different diagnostically relevant anatomical structures for T1 and T2 scans and eight structures for TOF scans (Table 2), as well as general image impression (GII) as a separate quality criterion.

**Table 1:** Definition of grading scale used for assessment of image quality.

Grade		T1 and T2	TOF
**1**	Poor image quality	Indistinct anatomical delineation with multi-contouring and disturbed internal structure; non-diagnostic, re-scanning necessary.	Indistinct anatomical delineation of vessels with multi-contouring and disturbed internal structure; non-diagnostic, re-scanning necessary.
**2**	Fair image quality	Suboptimal anatomical delineation with minor contouring but diagnostic anatomical definition; sufficient image quality with diagnostic limitations.	Suboptimal anatomical delineation of vessels with minor contouring but diagnostic anatomical definition; sufficient image quality with diagnostic limitations regarding main clinical diagnoses (stenosis, occlusion, aneurysms, and dissection).
**3**	Good image quality	Well delineated anatomical structures with good internal anatomy preservation; standard MR examination without diagnostic limitations.	Well delineated vessels with good anatomy preservation; standard MR examination without diagnostic limitations.
**4**	Excellent image quality	Very well delineated and contrasted anatomy between structures and excellent internal anatomy preservation; no diagnostic limitations: image quality better than required diagnostically.	Very well delineated and contrasted vessels and excellent anatomy preservation; no diagnostic limitations: image quality better than required diagnostically.

**Table 2:** Anatomical structures evaluated in image quality assessment for the different sequences.

T1	T2	TOF
Superior frontal gyrus	Superior frontal gyrus	Internal carotid artery
Precentral gyrus (hand area)	Precentral gyrus (hand area)	Median cerebral artery, M1 segment
Postcentral gyrus (anterior cortex hand area)	Postcentral gyrus (anterior cortex hand area)	Median cerebral artery, M2 segment
Insula, extreme capsule, and claustrum	Insula, extreme capsule, and claustrum	Median cerebral artery, M3 segment
Putamen	Putamen	Anterior cerebral artery
Cerebellar folia at the level of the superior cerebellar peduncle	Cerebellar folia at the level of the superior cerebellar peduncle	Posterior cerebral artery
Anterior commissure and the columns of the fornix	Anterior commissure and the columns of the fornix	Superior cerebellar artery
Frontal horn of lateral ventricles	Frontal horn of lateral ventricles	Posterior communicating artery
Septum pellucidum	Septum pellucidum	
Third cranial nerve	Superior cerebellar artery	
Fifth cranial nerve	Vessels in cerebrospinal fluid space close to fifth cranial nerve entry zone	

Without motion, it is important that iMOCO does not deteriorate the image quality, e.g. due geometry updates based on noise. Therefore, it was tested whether the difference in the grades with and without correction was statistical significant. The variation in the grades for the scans with and without correction was small (only a few grade 2 was given, otherwise all grade 3), and therefore a sensitive test procedure was used. For each image contrast, all individual paired structural grades for all subjects were treated independently, and McNemars test was used to test the null hypothesis that the P-value of grade 3 for scans -iMOCO equals the P-value of grade 3 for scans +iMOCO (only grade 2 and 3 needed to be considered, as they were the only grades given for the scans without motion).

For the scans with deliberate motion, more variation was present in the scoring. Here a paired t-test was used (SPSS 25) to test whether the difference in grades between scans with and without correction could be attributed to chance. Separate tests were performed for each image contrast, for the anatomical structure grades averaged for each subject, and for the general image impression. The null hypothesis was that the (across subject) mean difference of grades with and without correction equals zero.

## Results

**Fig 2:**
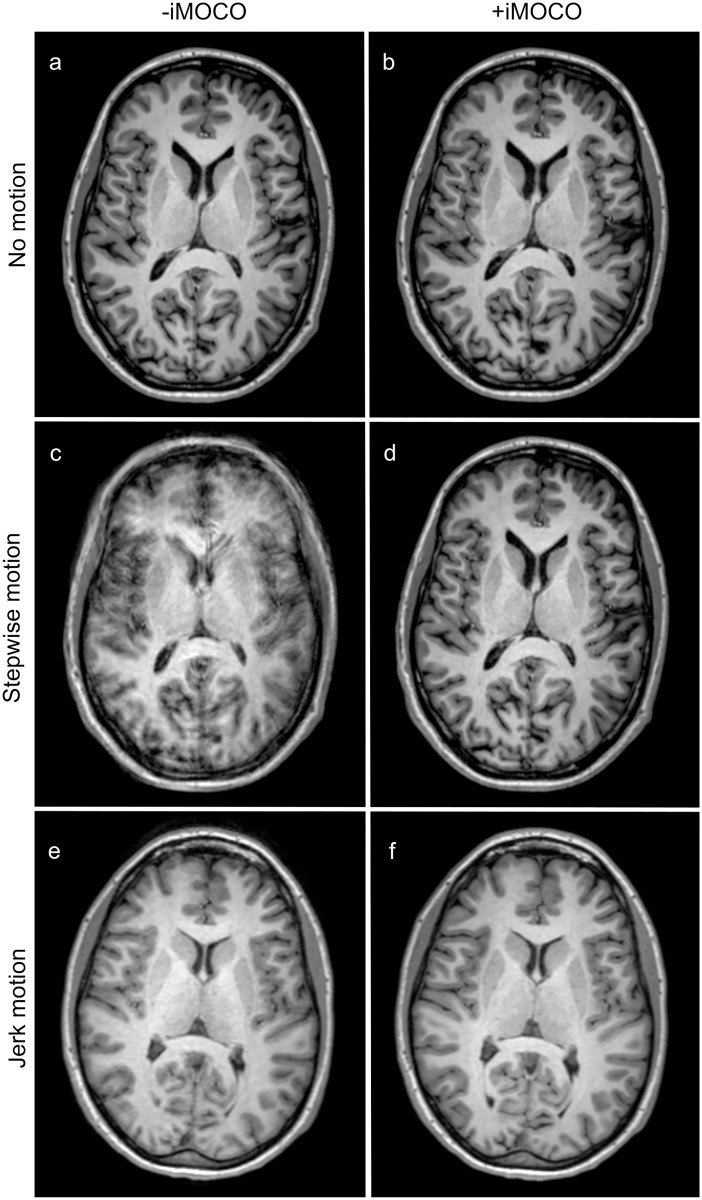
Examples of T1-weighted scans with (+) and without (-) iMOCO for different types of movement. Panels **a**‒**d** are from subject 1, while panels **e** and **f** are from subject 5. The scans in **a**, **b**, **d**, and **f** were given a grading of 3 in all quality criteria. For the scans with motion -iMOCO, panel **c** was given a grading of 1 in all criteria, while panel **e** was given a grading of 2 in all regions except for the frontal horns, the septum pellucidum, and the anterior commissure and the columns of the fornix (not illustrated), where grade 3 was given.

**Fig 3:**
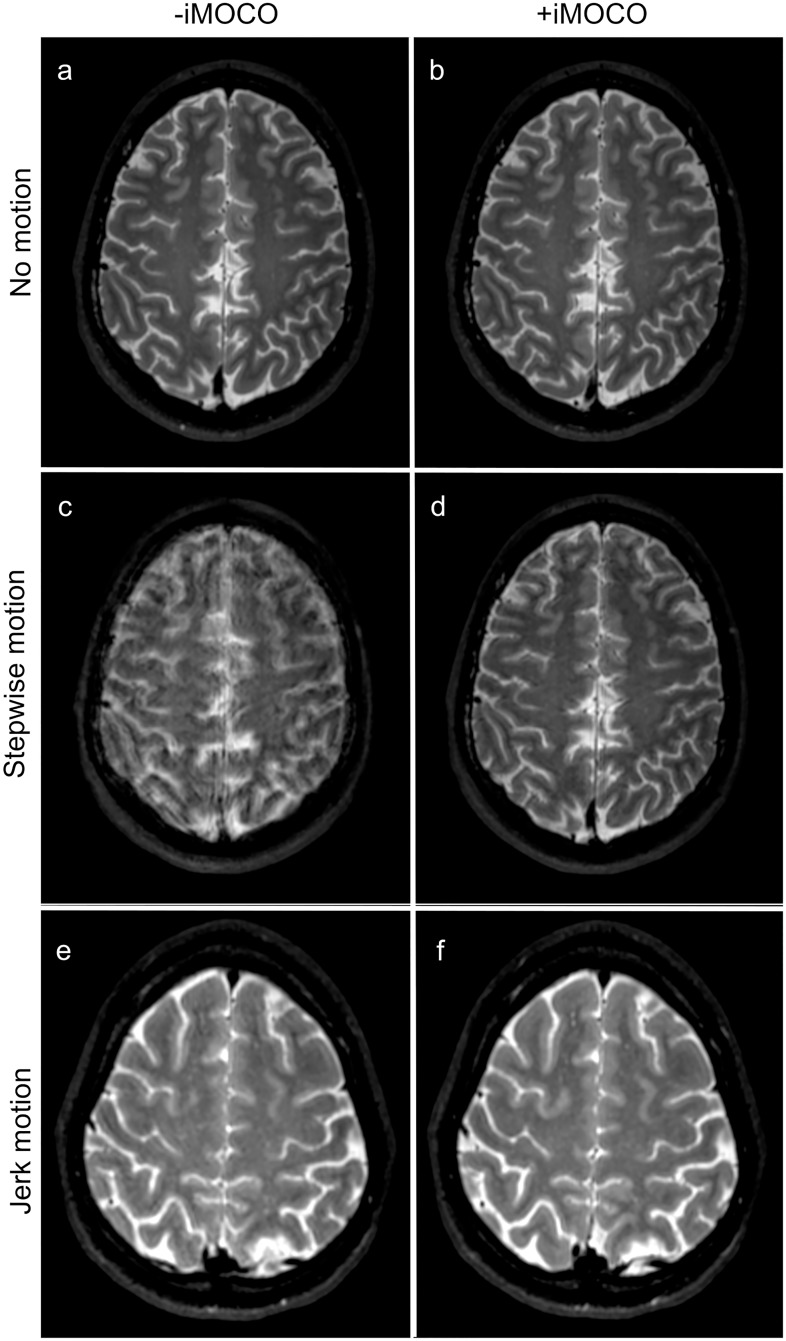
Examples of T2-weighted scans with (+) and without (-) iMOCO for different types of movement. Panels **a‒d** are from subject 1, whereas panels **e** and **f** are from subject 5. The scans in **a** and **b** were given a grading of 3 in all quality criteria except for two scored structures that are not illustrated (insula, extreme capsule, and claustrum; and putamen), which received a grading of 2. The scan in panel **c** was graded 1 in all criteria, whereas panel **d** was given a grading of 2 in all criteria except for frontal horn and septum pellucidum (both not illustrated), which were graded 3. With jerk motion, the artefacts were less problematic, and the scan represented in **e** was given a grading of 2 in all criteria while **f** was graded 3.

**Fig 4:**
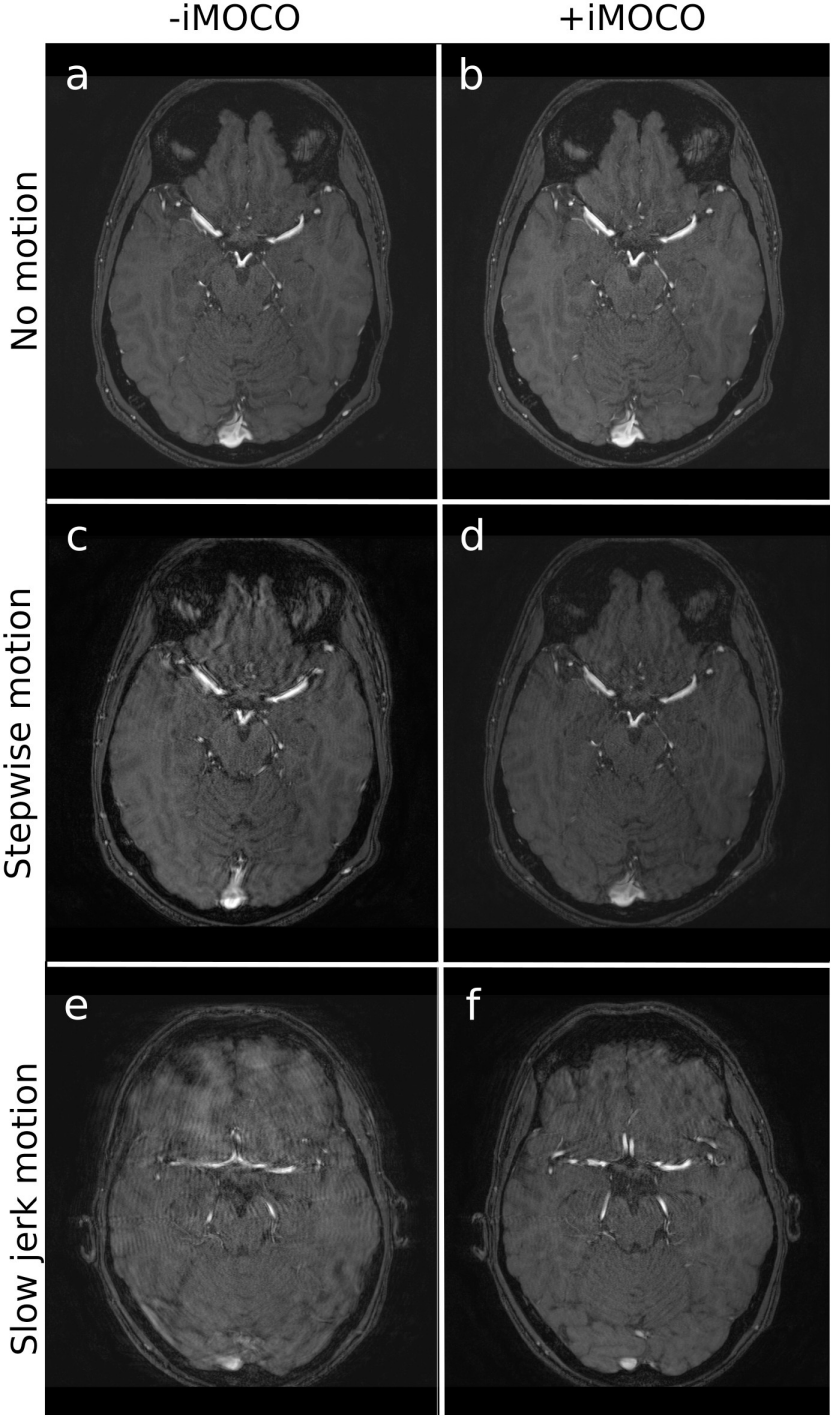
Examples of TOF scans with (+) and without (-) iMOCO for different types of movement. Panels **a**‒**d** are from subject 1, while **e** and **f** are from subject 7. The scans in panels **a** and **b** were given a grading of 3 in all quality criteria. The scans in **c** and **e** were performed with motion but -iMOCO, and were graded 1 in all criteria. The corresponding cases +iMOCO were given higher gradings, i.e. panel **d** was grade 3 in all criteria except general image impression (grade 2), and panel **f** was grade 2 in all criteria.

**Fig 5:**
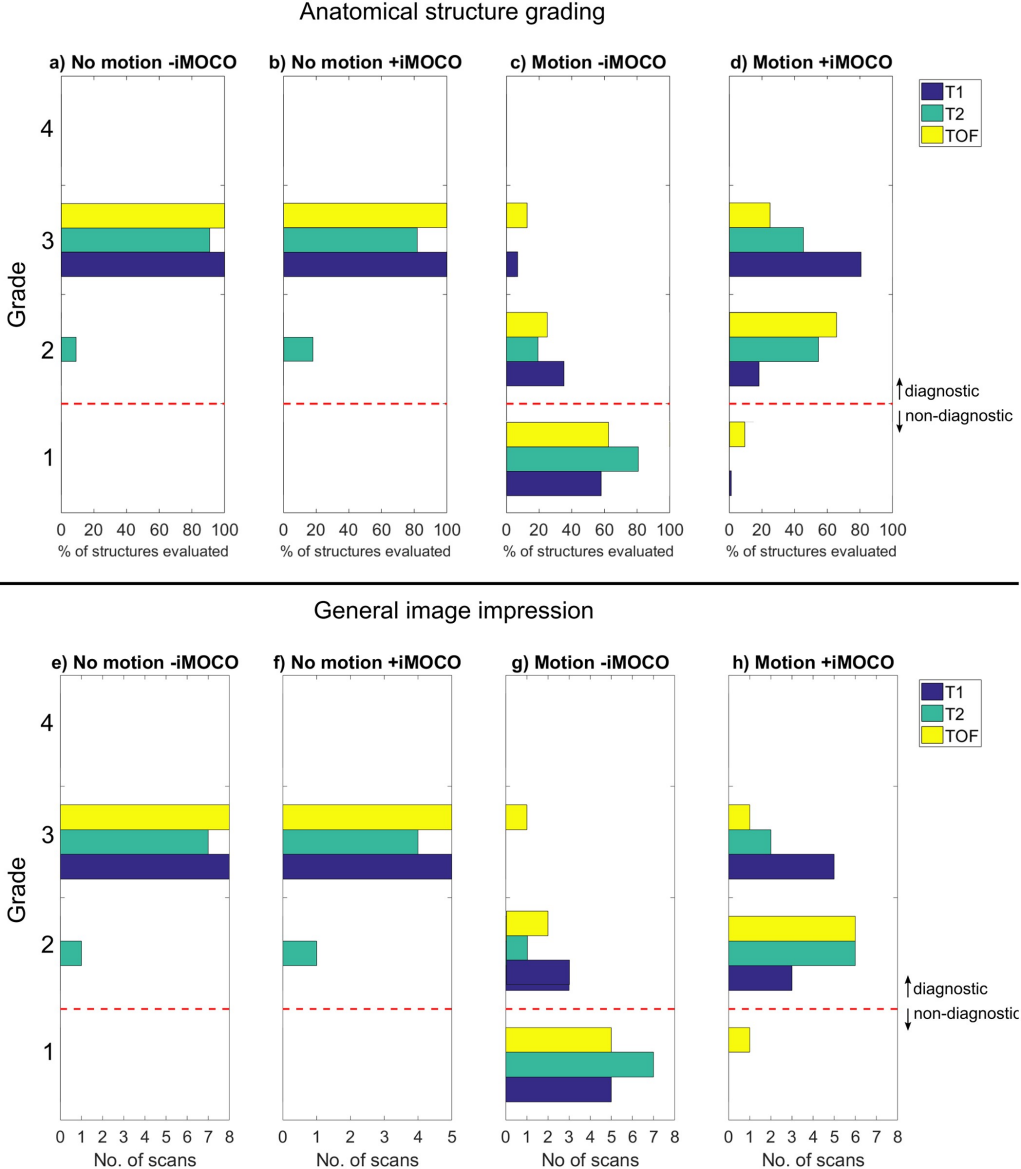
Summary of the grading of image quality. Panels **a**‒**d** show the pooled grades of all anatomical structures, given for the three types of sequence (T1, T2, and TOF), while panels **e**‒**h** show the general image impression (GII) grades for the three sequences.

Examples of image quality and grades are shown in Figs 2‒4 and 5 summarizes the grading results for all scans (all individual grades are in S1 File).

For scans with no deliberate motion, grade 3 was given for almost all structures and scans, and no significant difference in diagnostic image quality was observed between -iMOCO and +iMOCO scans. This was assessed for the three image contrasts separately. For the T1-weigthed and TOF scans, only grade 3 was given, and thus clearly no significant difference between–iMOCO and +iMOCO, see Fig 5a, 5b, 5e and 5f. For the GII grades for the paired T2-weighted scans, there was also clearly not a statistical significant difference in image quality between–iMOCO and +iMOCO, as in both cases 1 out of 5 received grade 2, and 4 out of 5 received grade 3. For the anatomical structure evaluations of the T2-weighted scans, 12 out of 55 anatomical structure evaluations were different between -iMOCO and +iMOCO, and in 8 of these 12 cases +iMOCO got the lowest grading (see Table 3). This corresponds to a P-value of 0.38 using McNemar’s test.

**Table 3:** Contingency table for the anatomical structure evaluations of the paired T2-weighted scans without motion.

	-iMOCO grade 2	-iMOCO grade 3	Row total
+iMOCO grade 2	2	8	10
+iMOCO grade 3	4	41	45
Column total	6	49	

For 5 subjects both–iMOCO and +iMOCO scans were performed without motion, and 11 structures were evaluated, leading to 55 pairs of grades. Only the grades 2 and 3 were given.

In scans with deliberate motion, there was a significant improvement in the image quality for scans +iMOCO compared to the scans -iMOCO in both general image impression (T1 p<0.01, T2 p<0.01, TOF p = 0.03) and in anatomical grading (T1 p<0.01, T2 p<0.01, TOF p = 0.01). The motion was not detected above the threshold in 18 of in total 309 movements of which it was reportedly not performed by the volunteer in 4 of those instances. The remaining 14 missed instances mainly occurred during fast jerk motion where the head was back to the original position in between navigator acquisitions.

With motion -iMOCO, image quality was severely deteriorated with GII graded as non-diagnostic in 91% of the scans, and with similar results for the anatomical structure grading (Fig 5c and 5g). However, in scans with motion +iMOCO the quality was dramatically improved, and GII was graded as diagnostic in 96% of the scans (i.e. 23 of 24 scans). The T1-weighted scans got the highest grading, which was almost as good as in the case of no motion (with motion +iMOCO, 81% of the structures evaluated were given a grading of 3, as compared to only 7% of the structures in the case of motion -iMOCO). TOF image quality was the most difficult to restore, as with motion +iMOCO 18% of the structures evaluated were graded 3, as compared to 9% with motion -iMOCO. However, the TOF examples still show clear diagnostic benefits from using iMOCO (Fig 4c–4f).

Different types of motion gave different degrees of artefacts in scans -iMOCO, with stepwise motion and slow jerk motion leading to the lowest grading, and jerk motion giving the highest (illustrated in Figs 2‒4). For all types of motion, +iMOCO scans received better gradings than the corresponding -iMOCO scans.

For each scan, most structures received the same grades (equal to the GII grade), but with exceptions, as illustrated in Figs 2‒4. Grade 4, corresponding to an excellent image quality that is better than diagnostically required, was not given at all.

**Fig 6:**
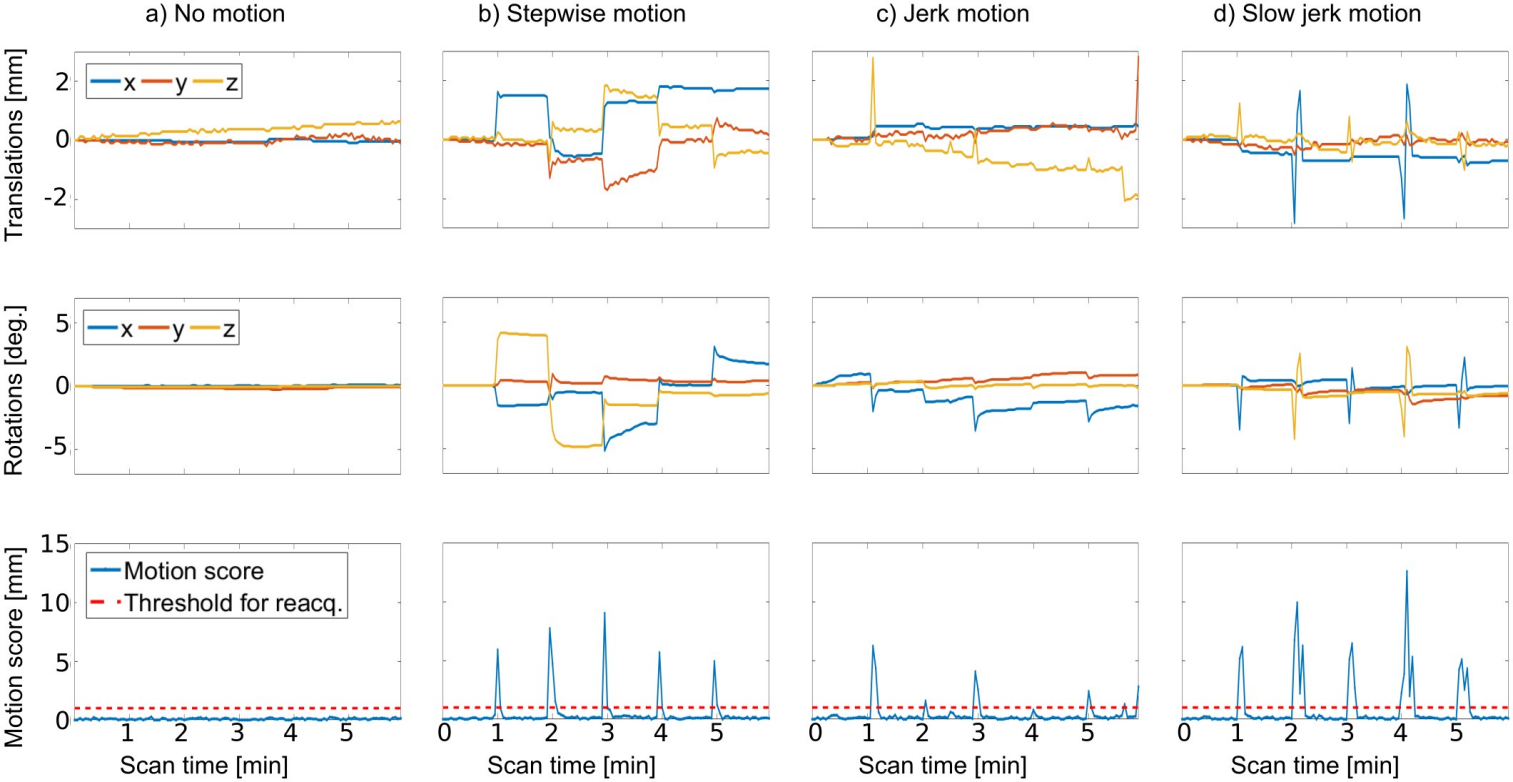
Examples of position parameters obtained from the navigators in the T1 sequence, for the different motion scenarios. The translations and rotations shown are the accumulated motion updates performed, and thus reflect the patient position relative to the starting position. The motion score reflects the change in position between every navigator and leads to reacquisition of the previously acquired k-space segment in the T1 sequence when above 1 mm.

Examples of motion monitored by the navigators with the different motion scenarios are shown in Fig 6. With jerk motion, some rapid jerks probably happened in-between two navigators, and were therefore not fully observed. Furthermore, especially in the subject doing jerk movements, a drift in the position over time occurred.

The accumulated motion scores during the scans are summarized in Table 4, as a measure of the amount of motion during each scan, and they were generally of the same order of magnitude in paired scans (-iMOCO and +iMOCO). Scans with motion +iMOCO had higher accumulated motion scores than corresponding -iMOCO scans in 18 out of 24 cases, but also longer durations due to reacquisition. With no motion, the T2 scan of subject 6 showed a remarkably high accumulated motion score for the -iMOCO scan. However, this scan received grade 3 in all quality criteria. The extent of motion in all scans ranged from 0.1 to 25.71 mm in maximum absolute displacement and from 0.2 to 15.0 degrees in maximum absolute rotation.

**Table 4:** Accumulated motion scores during all scans, and the relative increase in scan time for the scans with motion (MO) and correction (iMOCO).

Sequ-ence	Sub-ject	Type of move-ment	Accumulated motion score (mm)				Increase in scan time due to re-acquisition (%)
			no MO -iMOCO	no MO +iMOCO	MO -iMOCO	MO +iMOCO	
T1	1	Stepwise	10	10	58	71	10
	2	Stepwise	14	15	119	139	13
	3	Stepwise	22	18	71	51	3
	4	Stepwise	15	-	174	152	14
	5	Jerk	16	15	42	32	3
	6	Jerk	17	-	79	111	13
	7	Slow jerk	8	9	103	189	21
	8	Slow jerk	22	-	241	333	19
Average for all subjects			15	13	111	135	12
T2	1	Stepwise	20	20	97	102	10
	2	Stepwise	24	22	111	116	8
	3	Stepwise	32	62	126	127	5
	4	Stepwise	20	-	106	133	10
	5	Jerk	28	27	56	69	10
	6	Jerk	40	-	83	127	9
	7	Slow jerk	19	17	201	261	28
	8	Slow jerk	28	-	284	336	13
Average for all subjects			26	30	133	159	12
TOF	1	Stepwise	7	11	33	42	9
	2	Stepwise	5	6	54	58	8
	3	Stepwise	13	8	45	44	9
	4	Stepwise	5	-	48	45	5
	5	Jerk	12	11	91	83	20
	6	Jerk	5	-	66	161	44
	7	Slow jerk	4	8	99	134	40
	8	Slow jerk	11	-	43	87	12
Average for all subjects			8	9	60	82	18
Average for all subjects and sequences			17	17	101	125	14

Increase in scan time due to reacquisition in scans with motion +iMOCO ranged from 3% to 44%, with an average value of 14% (Table 3). Reacquisition occurred in one scan without deliberate motion +iMOCO with an increase in scan time of 0.8%. For all scan types, the (slow) jerk type motion clearly led to most reacquisitions, especially for the TOF sequence.

## Discussion

In this paper we have evaluated the improvements in diagnostic image quality for T1-weighted, T2-weighted, and TOF angiography sequences when using a prospective motion correction method, for different types of movement in healthy volunteers.

Image quality was estimated based on visual observation by a highly experience radiologist, and a four-step scoring system was used. Here, image quality of 1 indicates a non-diagnostic image quality, a grade of 2 indicated a sufficient or fair image quality, a grade of 3 indicated standard or good image quality and a grade of 4 indicated excellent or better than needed image quality. The grade of 4 was not given to any of the scored features, indicating that the image quality was not better than needed. For evaluating if the images were diagnostically useful we used the separation between grades 1 and 2 and above.

In the experiments without motion, the diagnostic image quality was graded as not being significantly different in scans with and without motion correction. This is an important evaluation with prospective motion correction techniques, as there is a risk of worsening of image quality in cases without motion if false geometry updates are applied, due to noisy motion parameter estimates.

In the experiments with movement, the evaluation clearly showed that motion correction significantly improved the diagnostic quality for all three sequences. For the T1-weighted sequence, the grading was almost as good as in scans with no motion. The more moderate grading of the TOF sequence was probably because the navigator takes 1.8 s to acquire, and movement during that period corrupts the navigator quality. The delay of 1.8 s from each navigator is completed until the update takes place, can also be responsible, however, the reacquisition is expected to remove the effect of the delay for large movements. The T2-weighted sequence, too, was not graded as good as the T1-weighted sequence. We speculate that the T2-weighted sequence is more sensitive to movement, due to the bright CSF signal giving rather strong ghost artefacts―even for motion below the reacquisition threshold. Lowering the reacquisition threshold would in that case lead to improvements, at the cost of a possible increase in scan time.

In the T1-weighted and T2-weighted sequences, we used navigators similar to the vNAV’s that has previously been demonstrated and evaluated for T1- and T2-weighted imaging [14,16]. The previously published evaluations of vNAV’s were mostly focused on the effects for brain morphometry estimation, but did not directly show evaluations of the *diagnostic* impact, which our evaluation is focused on. When inserting navigators into a sequence, care should be taken to minimize the effect on the steady-state magnetization, contrast, timing and duration of the target sequence. The originally proposed vNAVs [14] require around 500 ms of dead time in the sequence can only be used in sequences with long relaxation delays such as 3D magnetization-prepared rapid gradient echo (MPRAGE) [22] or spectroscopy sequences [23], but modified vNAV versions have been demonstrated e.g. in a FLASH sequence [24] which has no or very little dead time. For this FLASH application, the TRs and tip angles for the FLASH and vNAV were matched to ensure the steady state of the FLASH sequence was not disturbed by the vNAV. Our navigator incorporation into the TOF sequence was different, as we used a short TR, low tip angle, fat selective, non-EPI, segmented navigator, to not disturb the water magnetization, and the steady-state eddy currents of the TOF sequence, which has highly suppressed water signal. Retrospective motion correction of TOF sequences using similar segmented fat navigators has recently been demonstrated in a conference contribution [25]. Compared to prospective techniques, retrospective motion correction has the drawbacks that spin-history effects are not compensated, rotations can lead to local Nyquist violations in k-space, and reacquisition cannot be used [4]. On the other hand, the prospective corrections of TOF in our work have a delay of 1.8s, to not disturb steady state of the TOF. Whether retrospective (without delay, but with the limitations of retrospective techniques) or prospective correction (with a delay) leads to the best image quality, remains to be shown. We believe that our prospective technique will be superior to the retrospective technique with highly uncompliant subjects, as large movements are generally difficult to compensate for with retrospective techniques.

Since there is only one motion update for each navigator volume acquired, the update rate of iMOCO is relatively slow compared to camera systems which, on the other hand, require specialized hardware and challenge patient handling. At this point, reacquisition was used to correct for the corrupted data in-between two updates. Reacquisition, however, increases scan time depending on the time interval of geometry updates, the reacquisition threshold, and the amount and type of motion. In our experiments, the “slow jerk” motion clearly led to most reacquisition, because the slow movement meant that the time that the subject was still during a scan was least. The TOF sequence had the highest density of motion because of the short scan duration, and that is probably the reason for the TOF with “slow jerk” motion leading to the highest relative increase in scan time. The fast jerk type motion was missed in 14 movements when the motion occurred in between two navigator scans and the head was back in the original position before motion was detected, this is an inherent limitation to the low temporal resolution of navigator based motion correction.

Unfortunately, when evaluating prospective correction, an uncorrected scan cannot be retrieved from the same data. Thus, separate scans were acquired to obtain scenarios with and without correction, where the subject might have moved differently. Despite receiving careful instructions, it is impossible for a subject to reproduce motion in scans with and without correction, which is also reflected in Table 4. However, in our comparison, at least we did not favour the scans with correction―as in most cases these had higher accumulated motion scores than corresponding scans without correction. The scans were acquired in healthy volunteers, but by using different types of movement and different degrees of motion, we attempted to cover the diverse types of movement in a patient population. However, evaluation of the method in a patient population would be an interesting future study.

In conclusion, we have shown significant improvements in the diagnostic quality of structural and angiographic MRI during subject movement.

## Supporting information

S1 File Grading results for all scans and structures. (XLSX)
